# Compressive Sensing of Roller Bearing Faults via Harmonic Detection from Under-Sampled Vibration Signals

**DOI:** 10.3390/s151025648

**Published:** 2015-10-09

**Authors:** Gang Tang, Wei Hou, Huaqing Wang, Ganggang Luo, Jianwei Ma

**Affiliations:** 1 School of Mechanical and Electrical Engineering, Beijing University of Chemical Technology, Beijing 100029, China; E-Mails: tanggang@mail.buct.edu.cn (G.T.); zzhouhou@163.com (W.H.); luo93_buct@hotmail.com (G.L.); 2 Beijing Zhongfang Jingye Electromechanical Device Co., Ltd., Beijing 101111, China; 3 Department of Mathematics, Harbin Institute of Technology, Harbin 150001, China; E-Mail: jma@hit.edu.cn

**Keywords:** roller bearing, fault detection, compressive sensing, harmonic detection, matching pursuit

## Abstract

The Shannon sampling principle requires substantial amounts of data to ensure the accuracy of on-line monitoring of roller bearing fault signals. Challenges are often encountered as a result of the cumbersome data monitoring, thus a novel method focused on compressed vibration signals for detecting roller bearing faults is developed in this study. Considering that harmonics often represent the fault characteristic frequencies in vibration signals, a compressive sensing frame of characteristic harmonics is proposed to detect bearing faults. A compressed vibration signal is first acquired from a sensing matrix with information preserved through a well-designed sampling strategy. A reconstruction process of the under-sampled vibration signal is then pursued as attempts are conducted to detect the characteristic harmonics from sparse measurements through a compressive matching pursuit strategy. In the proposed method bearing fault features depend on the existence of characteristic harmonics, as typically detected directly from compressed data far before reconstruction completion. The process of sampling and detection may then be performed simultaneously without complete recovery of the under-sampled signals. The effectiveness of the proposed method is validated by simulations and experiments.

## 1. Introduction

Roller bearings are integral for ensuring the security and stable operation of mechanical systems with rotating machinery components. Critical consequences may result once bearing failure occurs, which may be far worse in the absence of adequate monitoring. Proper monitoring for running conditions of roller bearings is then essential to guarantee the safe operation of rotating machinery.

Bearing fault diagnosis has been studied over the past decades. Abundant status information can be consistently obtained from vibrations, making vibration signal analysis a common and effective bearing diagnosis method. Vibration signal analysis may be performed typically in the time domain, frequency domain or time-frequency domain [1,2]. Statistical parameters are adopted to detect and predict bearing faults in the time domain. This method type is easily implemented, though it cannot distinguish fault types with high precision [3]. Frequency analysis is also applied to extract fault features, e.g., fault frequencies. Envelope analysis is a frequency analysis method [4,5], that identifies fault types by highlighting characteristic fault frequencies in a spectral domain. Signals acquired by sensors, however, are often mixed with noise, adding difficulty for effective fault features extraction. Time-frequency methods are developed to solve these issues including empirical mode decomposition [6,7,8] and wavelet analysis [9,10], and are generally based on the Shannon sampling theory that sample frequency must be twice the maximum frequency. The theory indicates that a large amount of data must then be collected, creating an exceptional challenge for signal acquisition, transmission and processing. Compression of large-scale monitoring data to detect fault features directly from sparse samples is one way to address the challenges. According to the theory of compressive sensing, a signal may be reconstructed from under-sampled linear measurements. The theory represents a significant breakthrough in the signal processing field and has attracted a great deal of attention since its proposal. Compressive sensing has been widely applied over time in various fields, e.g., magnetic resonance imaging [11], seismic wave processing [12], yet many of these studies have been associated with signal or image reconstruction.

For the roller bearing fault detection, running information can be extracted with well-designed sampling, where fault feature detection from sparse samples is possible by implementing the correct strategy. Bearing fault features may also often be identified far before completion of the reconstruction of under-sampled signals, thus it is not necessary to recover a signal perfectly for fault diagnosis. The effectiveness of statistical inference based on compressive sensing has been verified in references [13,14,15], suggesting the possibility that some characteristic parameters may be estimated from only a few compressed measurements without ever recovering the actual signals. Sparse event detection strategies based on compressive sensing have since been extensively explored in related fields, such as wireless sensor networks [16] and has also been deemed attractive in the field of machinery fault diagnosis. Li attempted to compress and reconstruct monitoring data of a train rolling bearing [17]. Chen built a learning dictionary frame to extract a fault-impact signal [18]. Zhang performed a preliminary study on compressive detection issues of bearing faults [19]. Tang developed a sparse classification method for rotating machinery faults based on compressive sensing strategy [20]. The results of these studies validate the effectiveness of compressive sensing in machinery fault diagnosis; however, their focus was primarily on sparse representation or reconstruction of fault signals.

Accurate extraction of bearing fault features from the compressed vibration signals remains an obstacle. The focus of this paper then is on development of an applicable detection strategy for roller bearing faults from under-sampled signals and performance of the sampling and detection simultaneously without complete signal reconstruction. Statistical inference based on compressive sensing has been studied in other fields [13,14,15] as mentioned above, yet there are still many obstacles to surmount when applied to bearing fault detection. The bearing fault signal consists of impulses and in the commonly utilized Fourier or wavelet domain, its sparsity does not completely meet the requirements for compressive sensing, increasing the difficulty of the compressive sensing process. Remaining to be resolved also is the identification of bearing fault features to be extracted from under-sampled signals and the process for integrating compressive sensing into the bearing fault diagnosis technique. The research in this study involves the development of an applicable monitoring strategy for bearing faults from under-sampled vibration signals, and the simultaneous performance of sampling and detection without a complete recovery of the incomplete signal.

Harmonic resonance typically occurs for rotating machinery when a roller element with defects strikes another bearing surface. Some characteristic harmonic resonance then is selected as a bearing fault indicator and a compressive sensing strategy of roller bearing faults via characteristic harmonic detection is then developed. During the process of incomplete reconstruction, an orthogonal matching pursuit strategy is employed to search for characteristic harmonic waves among sparse samples. The sparsity of potential fault signals may be deduced from *a priori* knowledge, and then harmonic components related to fault features may be detected through matching pursuit. Fault features may typically be detected far in advance of perfect reconstruction completion, thus allowing fault detection to be achieved from only a miniscule amount of compressed data. To our knowledge, research related to compressive sensing of bearing fault via characteristic harmonic detection has not been addressed in the past, rendering this study unique.

A brief overview of this paper is as follows: Section 2 provides a description of bearing fault detection issues; Section 3 briefly introduces the proposed strategy, including a bearing fault indicator of harmonic resonance, compressive sensing of the indicator and the compressive fault detection scheme. Section 4 explains and verifies flexibility of the proposed strategy through several simulations. Section 5 illustrates the experimental results with applications to typical roller bearing faults. Section 6 concludes the analysis.

## 2. Problem Statement

Similar to common signal detection in most fields, without loss of generality, a simple detection issue of bearing faults may be formulated as:

*H0*: *x* = *w* + *n*

(1) *H1*: *x* = *s* + w + *n*

where *s* is a known signal of interest, *n* is *i.i.d.* Gaussian noise, *x* denotes the observation signal. Generally, besides the noise *n* and a potential fault signal *s*, the acquired data *x* is often mixed with interference signals *w* from devices around.

Provided *s* denotes a vibration signal related to a bearing fault, the fault detection problem then is to distinguish the hypotheses between *H0* and *H1*. If the hypothesis *H1* is true, then a fault may exist in the bearing.

Various methods have been developed to distinguish the fault component *s* from the mixture signal *x*. One commonly utilized method is to proceed in a transform domain:

(2) $$H1:y=\Phi x=\Phi \varphi^{H}\varphi x=\Phi \varphi^{H}\varphi (s+w+n)=A\varphi (s+w+n)$$

where *x* is a *N* × *1* vector signal, Φ is a *M* × *N* measurement matrix, *M* ≤ *N*, and each row of Φ represents a sensor to measure *x.* φ is a *N* × *N* column orthonormal basis matrix, the superscript *H* denotes a conjugate transposition. $A=\Phi \varphi^{H}$ is often designated the sensing matrix to measure the transformed data *u* = φ*x.* *y* is a *M* × *1* measurement vector denoting the observation of *y* = *Au.*

Most observations are under the limitation of Shannon principle, requiring full samples, or at least adequate data. When all *N* measurements are available, *i.e.*, *M* = *N*, then, $\Phi^{H}\Phi \;=\;\Phi \Phi^{H}\;=\;I_{N\times N}$, indicating *y* is an observation of *x* with full sampling. Especially, if Φ = *I*, then, *y* = φ*^H^u* = φ*^H^*φ*x* = *x*, where *u* = φ*x* indicates a decomposition of *x* in transform bases φ*.*

The case of *M* << *N* is often encountered or expected to relieve pressure of data acquisition and limitations due to incomplete and imprecise knowledge. *y* is then indicated as a compressive sensing of signal *x*. It would be promising if some required information of original signal *x* can be deduced from the compressed observation *y* without reconstruction, *i.e.*, compressed detection problem. Compressive sensing is introduced to solve the compressed detection problem in other research areas mainly related to simple detection problems as stated in Equation (1) [13,14] and assuming the component *w* as a narrowband interference. Considering the complex characteristic of bearing fault signals, many obstacles must be overcome.

## 3. The Proposed Strategy

### 3.1. Characteristic Harmonics Acting as an Indicator to a Bearing Fault

Local defects existing in a bearing will cause the fault of a bearing surface striking another and an impulse force to be produced at a certain period, setting off a series of high-frequency damping vibrations. The frequencies generated from periodicity impulsions are referred to as bearing fault frequencies or fault characteristic frequencies and may be theoretically calculated if the working conditions are given. Application of the envelope demodulation to the high-frequency damping vibration signals allows one to separate periodic damping vibrations and the fault features to then be extracted. Bearing outer race faults, for instance, retain a series of discrete spectral lines in the envelope spectra at intervals of every ball pass frequency, with magnitudes decreasing gradually in sequence [21].

The signal *s*, in Equation (2) may then be described as a combination harmonics series:

(3) $$s(t)=A_{0}cos(2\pi \omega_{0}t+\theta_{0})+\sum_{n=1}^{L}A_{n}cos(2\pi \omega_{n}t+\theta_{n})$$

where $A_{0}$, $\omega_{0}$ and $\theta_{0}$ denote the amplitude, frequency and phase of the fundamental wave, respectively. Assuming there are *L* + *1* harmonic waves, $A_{n}$, $\omega_{n}$ and $\theta_{n}$ denote the amplitude, frequency and phase of the *n-th* harmonic wave, respectively.

Equation (2) then becomes:

(4) $$H1:H(y)=Au=A\varphi [A_{0}cos(2\pi \omega_{0}t+\theta_{0})+\sum_{n=1}^{L}A_{n}cos(2\pi \omega_{n}t+\theta_{n})+w+n]$$

If we assume φ denotes the Fourier operator F, then the spectra of the mixed signal *x* may be observed as:

(5) $$\begin{array}{c} u=F[A_{0}cos(2\pi \omega_{0}t+\theta_{0})+\sum_{n=1}^{L}A_{n}cos(2\pi \omega_{n}t+\theta_{n})+w+n]\\ \text{=}{\tilde{A}}_{0}\omega_{0}\text{+}\sum_{n=1}^{L}{\tilde{A}}_{n}\omega_{n}\text{+}F(w+n)\text{+}C \end{array}$$

where ${\tilde{A}}_{0}$ and ${\tilde{A}}_{n}$ denote the corresponding amplitudes of frequency $\omega_{0}$ and $\omega_{n}$, C is a constant. The interference components *w* and *n* are typically not harmonic.

If a fault exists in the bearing as observed in Equation (5), several characteristic harmonics will be aroused and a characteristic frequency may be detected. If the resonant harmonics can be detected from only a few samples of *x* for a compressed observation data *y*, then the compressive sensing of bearing faults will be solved.

### 3.2. Detection of Harmonic Resonance from Compressed Samples

Provided a perceptual measurement matrix, as stated above, $A=\Phi \varphi^{H}$ satisfies the constraint conditions of isometric, u=φx, then define a representation of a sparse signal *x* as:

(6) y=Au

Provided *x* is composed of only resonant harmonics *s*, which are related to a bear fault, the projection of *s* onto the measurement vector may be formulated as:

(7) $$\begin{array}{ccc} \min{\left\|s\right\|}_{0} & s.t. & y=Au=\Phi \varphi^{H} \end{array}(\varphi s)$$

Let *K* denote the sparsity of the harmonic signal *s* in a pre-defined transform domain φ; ensure the energy of the *K* elements in vector *y* is approximately equal to that of the corresponding *K* elements in vector *u* and the vector *s*. Particularly, the maximum *K* sparse components of *y* are approximately equal to the corresponding maximum *K* elements of *u*, respectively, where *y* is a sparse representation of the sparse signal *y*. Detection of resonant harmonics from spectral energies is now the challenge. The first several harmonics are typically adequate to determine the presence of a bearing fault and the harmonic property of the interference components *w* and *n*, allow the method to also work for a mixed signal *x* = *s* + w + *n.*

Initially, the case of compressed sampling, *i.e.*, *M* << *N* for sensing matrix *A* is mainly considered, as mentioned in Section 2. The detection issue then is to pursue the first several resonant harmonics with high energies from the undetermined problem (Equation (7)). A strategy of Compressive Sampling Orthogonal Matching Pursuit (*CoSaMP*) [22] is employed to solve the undetermined equation and to pursue the resonant harmonics related to bearing faults.

Inspired by a tolerance constraints isometric condition, the *CoSaMP* algorithm is developed to search for the signal location’s largest element and to derive a perfect reconstruction of the signals *u* and *x* by iterative optimization. Several best matched atoms to the signal are selected from a measurement matrix *A* to construct a sparse approximation for a signal in each iteration. Redundancy errors exist in each iteration between the previous and the current approximate values containing potential components that have not been extracted from the signal. The redundancy errors will then be updated and a new redundancy signal representation generated with the maximum component of the current signal identifiable through matching pursuit. The next iteration follows until the redundancy errors become adequately reduced and the signal *u* may be linearly represented by the selected atoms. Accurate reconstruction of a sparse signal is possible with the measurement matrix criteria satisfaction of select criteria.

The sparsity *K* of a signal should be known *a priori* for most existing compressive sensing reconstruction algorithms; however, in practice, the sparsity *K* cannot be obtained directly in most cases, so *K* must then be estimated, increasing the possibilities for reconstruction errors in the compression sensing frame process. If the sparsity cannot be estimated or is inaccurate, an obstacle to the application of compressive sensing will exist.

Complete reconstruction of a signal is not necessary in all cases. The vibration signal of a roller bearing, for example, consists of many sub-components. If the sparsity *K* of a sub-component is known, then it can be identified with the aid of matching pursuit, so if a sub-component is related to fault features and its sparsity *K* is known, the sub-component is detectable without complete reconstruction. The proposed detection strategy in this paper is based on this principle.

### 3.3. Compressive Sensing Scheme of a Bearing Fault via Characteristic Harmonic Detection

A bearing vibration signal typically contains periodic impulses and is not adequately sparse in the Fourier domain, exerting a negative effect on a perfect signal reconstruction. Components are not all closely related to fault features; however, and if the fault features are detected in advance, the process for complete and perfect reconstruction may be terminated prematurely.

**Figure 1:**
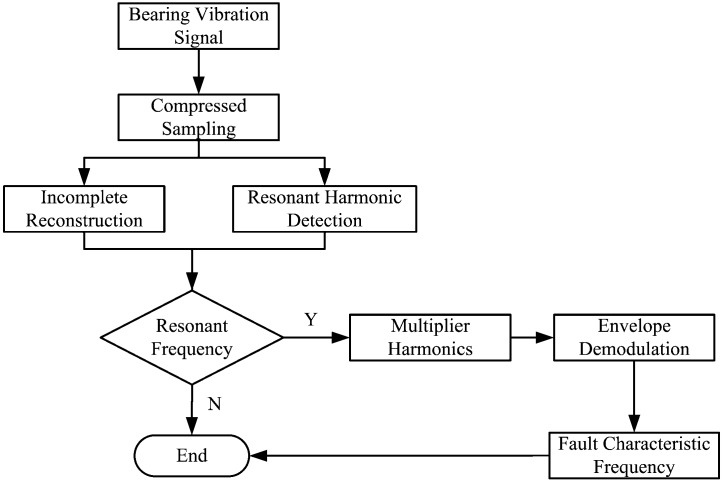
Scheme of the proposed strategy.

The sparsity *K* of a bearing vibration signal is often difficult to estimate, yet a harmonic wave sparsity *K* = *2* in Fourier domain is well-known and a bearing vibration envelop signal often contains harmonic waves related to the fault features. According to the matching pursuit strategy then, if the sparsity *K* = *2*, then the harmonic component in the signal with maximum energy may be detected. The possibility exists then to detect harmonic components related to fault characteristic frequencies, multiplier harmonic frequencies or their sidebands of a faulty bearing, indicating whether a fault exits in a bearing and determining the fault types. Characteristic harmonic detection is a reliable method for compressive sensing of roller bearing faults. The proposed strategy scheme is presented in Figure 1.

## 4. Simulation of Vibration Signals Induced by Bearing Faults

Vibration signal of a rolling bearing induced by a single outer-race fault may be simulated as follows:

$$x(t_{n})=A(t_{n}){\text{e}}^{-2\pi f_{n}\xi (t_{n}-{\text{T}}_{k})}*\sin(2\pi f_{n}(t_{n}-{\text{T}}_{k})+\phi_{0}),\;n=0,1,2,\ldots ,N-1$$

$$T_{k}=T_{k-1}+\Delta T_{k},\;k=1,2,3,\ldots$$

where {*A*(*t*_n_), *n = 0*, *1*, *2*, *…*, *N* − *1*} denotes the amplitudes of transient responses, often determined by the rotation speed, load distribution, fault size, fault location and other unknown factors in complicated manners. *f*_n_ = 3000 Hz is the resonance frequency of the system, ξ = 0.1 is the relative damping ratio, $\phi_{0}$ = 5 *rad* is the initial phase angle, $T_{k}$ denotes the trigger time of the *k-th* impulse, $\Delta T_{k}$ = 0.01 denotes the interval time between *(k−1)-th* and *k-th* impulses. The sampling frequency is 10 kHz.

**Figure 2:**
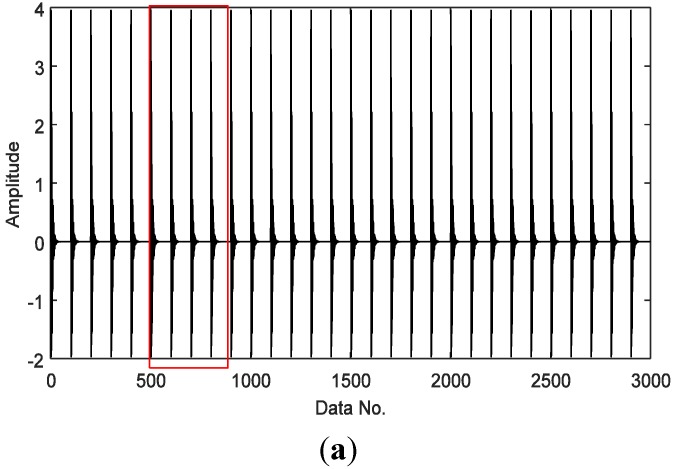

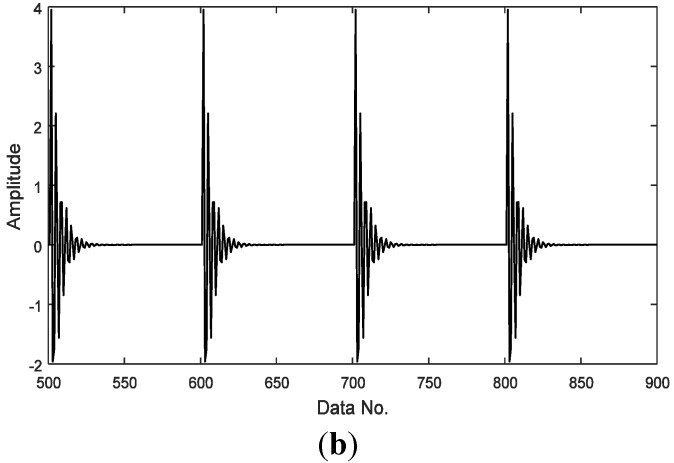
A simulated vibration signal induced by (**a**) outer race faults and (**b**) a typical section with four cycles within the red box in (**a**) is shown from zoom-in view.

**Figure 3:**
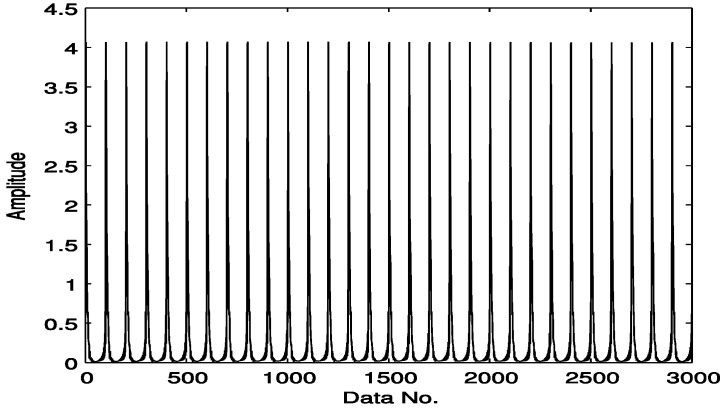
Envelope of the signal as shown in Figure 2.

A simulated vibration signal with outer race faults is shown in Figure 2. The envelope of the signal is obtained by envelope demodulation with the proposed strategy (Figure 3). Most processing approaches require a signal with full samples as an input, e.g., the signal as shown in Figure 2. The proposed technique in this paper is trying to reduce the amount of data acquisition and make it possible to detect fault features from under-sampled signals.

**Figure 4:**
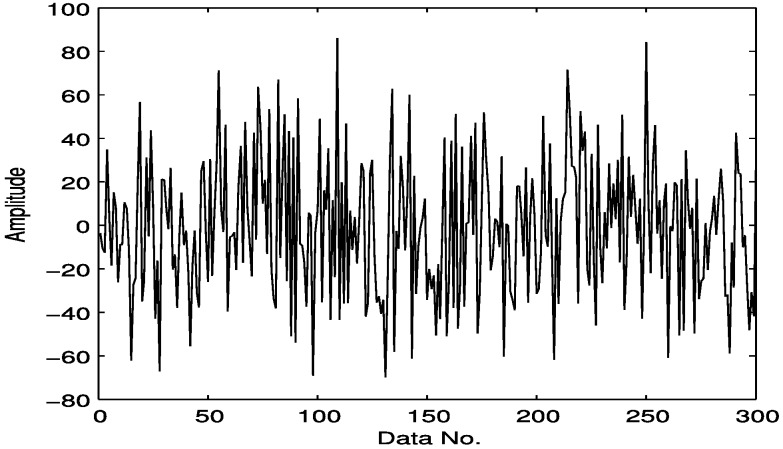
10% samples of the signal as shown in Figure 3.

Here we select a compressively sampled signal section from that of Figure 3, which is presented in Figure 4, with 300 samples compared to 3000 in Figure 3, *i.e.*, a sampling rate of 10%. Here the sampling is not in a traditional manner. Instead, to meet the requirements of compressive sensing as expressed in Equation (7), the original envelop signal as shown in Figure 3 is multiplied by a Gaussian random matrix with reduced dimension, which totally changes the sample size and the amplitude range of the original signal. Fortunately, it does not affect the detection effect, because here we mainly use the frequency features.

**Figure 5:**
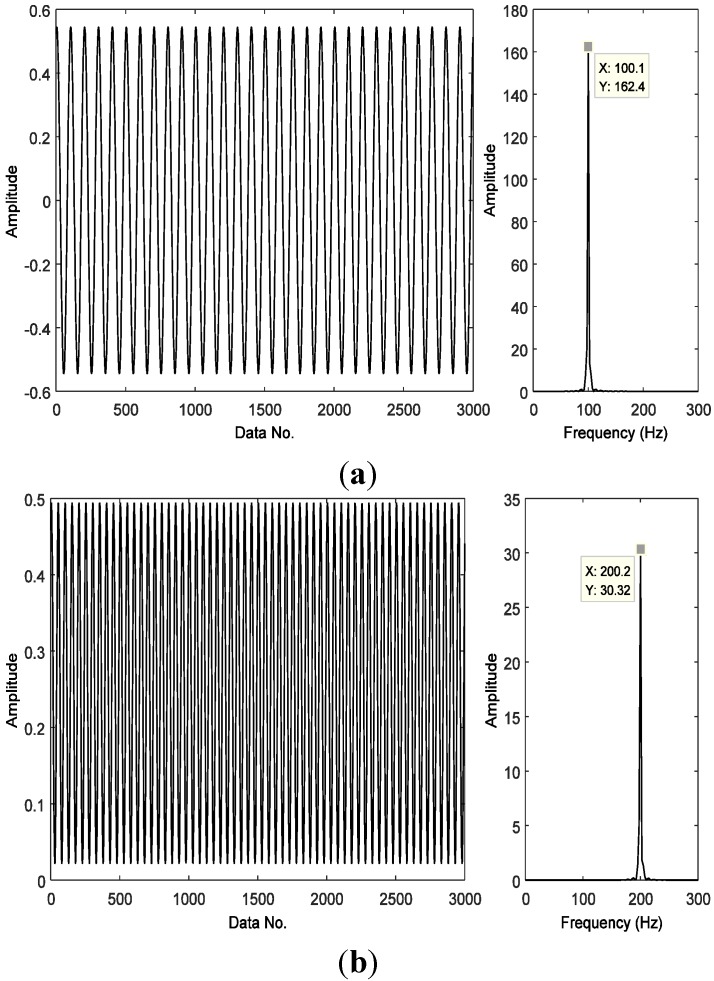
The first two detected harmonic components and their frequencies: (**a**) the first harmonic and its frequency; and (**b**) the second harmonic and its frequency.

The under-sampled signal then is taken as the input data for our method, instead of using full samples. Next, we try to detect the fault features during a reconstruction process. Most traditional methods often begin the detection process after the reconstruction is completed, *i.e.*, taking full samples of the recovered signal as the input. However, in our proposed technique, the fault features can usually be detected far before the reconstruction is completed, e.g., when only 30% of the recovery process is completed. Then the detected harmonic waves and their frequencies are recorded. That is to say, harmonic components corresponding to characteristic frequencies may also be detected from only a few sparse samples. The first and the second harmonic components detected are displayed in Figure 5 with the first harmonic corresponding to the fault frequency, and the frequency of the second harmonic equal to twice the fault frequency, confirming a bearing fault exists in the outer race.

## 5. Experiments

**Figure 6:**
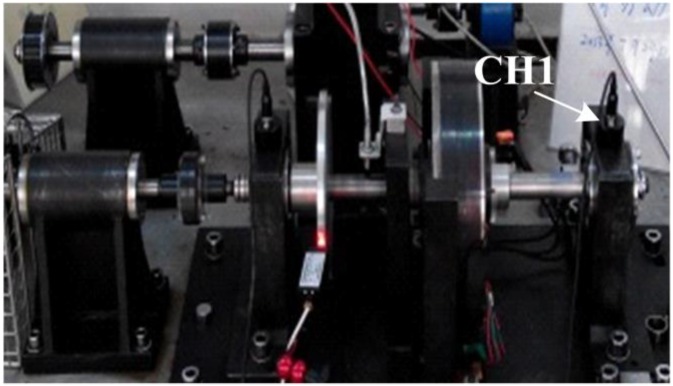
Experiment platform with fault rigs of a roller element bearing.

Several experiments are performed with fault rigs of roller element bearings to verify the effectiveness of the proposed method. The rig is composed of a motor, a coupling, a rotor and a shaft with two roller bearings (Figure 6). Four common cases are studied, including a perfect bearing, a bearing with an outer race defect, a bearing with an inner race defect and a bearing with a ball element defect. Utilizing an electron-discharge machining with a fault width of 7 mm and depth of 25 mm, a single point defect is individually introduced in the inner raceway, outer raceway and ball element of different bearings.

Vibration sensors are located at positions near the bearings to mitigate the effects of signal attenuation. The bearing housing is considered a superior location for bearing arrangement and is also utilized for the placement of vibration sensors in this work. Vibration signals are measured by an accelerometer located at the top of the bearing house (CH1, Figure 6). Sample frequency is 100 kHz and the shaft speed is a finite 1300 rpm for all study experiments. The sampling frequency is decreased to 5 kHz by a down-sampling post processing strategy for simplification purposes to illustrate the proposed strategy. A section of the post-processed signal with a few samples, e.g., 2000, is then selected as the object signal.

### 5.1. Outer Race Fault Detection of a Roller Bearing

**Figure 7:**
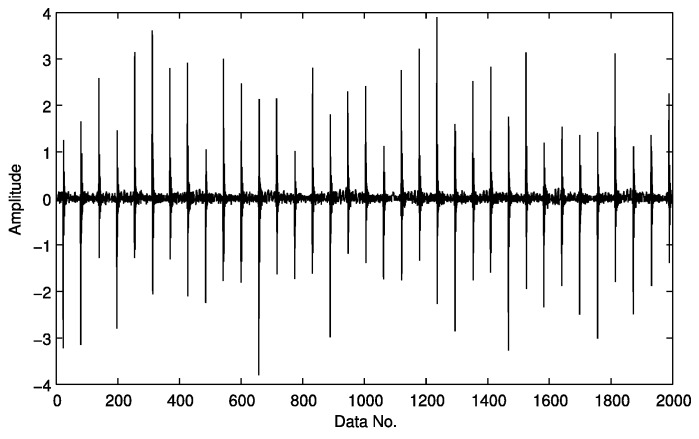
The vibration waveform of a bearing with an outer race fault.

A roller bearing with an outer-race fault is first created and a section of the post-processed signal with 2000 samples then selected as the object signal. Its calculated theoretical frequency is 86.33 Hz. A bearing vibration waveform with an outer race fault is displayed in Figure 7. Envelope demodulation is applied to the object signal for the purpose of detecting the fault features from only 200 compressed data samples.

**Figure 8:**
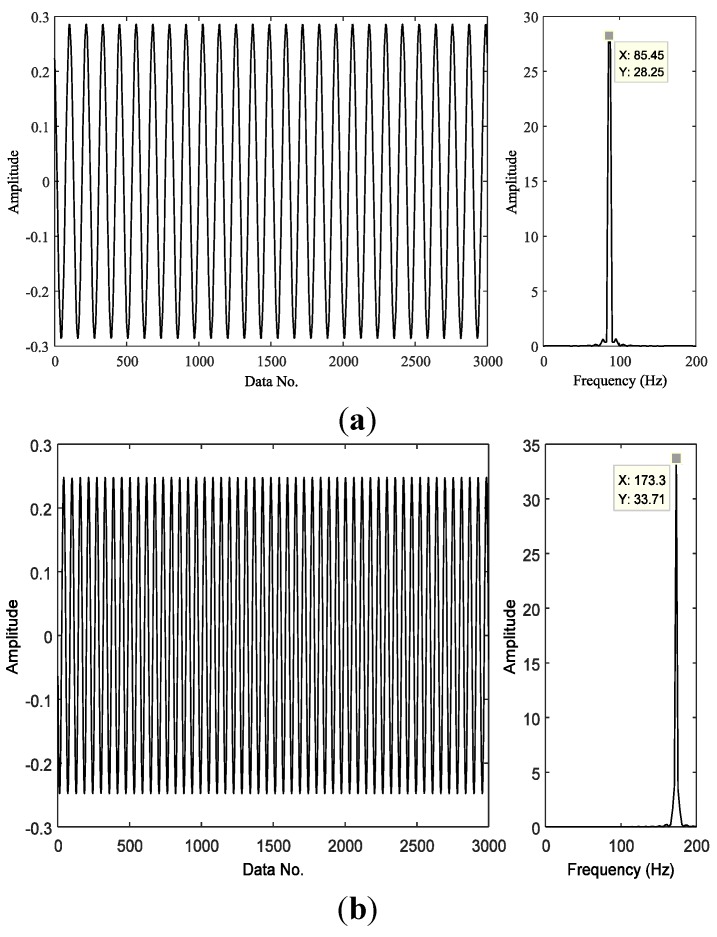
Harmonics detected and their frequencies from envelope samples of the signal shown in Figure 7: (**a**) the first harmonic and its frequency, and (**b**) the second harmonic and its frequency.

Assuming sparsity of the first potential harmonic is *K* = 2, the first harmonic and the second component may be detected from the compressed samples (Figure 8). The frequency of the first detected harmonic component is 86 Hz, similar to theoretical frequency of the outer race fault. The frequency of the second detected harmonic component is 172 Hz, twice the outer race fault frequency, indicating the presence of an outer race fault. 

### 5.2. Inner Race Fault Detection of a Roller Bearing

**Figure 9:**
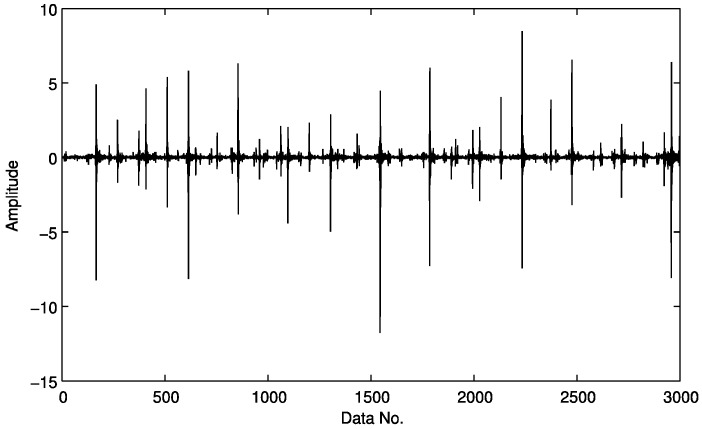
Vibration waveform of a bearing with an inner race fault.

Experiments with a bearing inner race fault are also performed to verify the effectiveness of the proposed method, and its calculated theoretical frequency is 145.83 Hz. A section of the post-processed signal is first selected with 3000 samples as the object signal. A bearing vibration waveform with an inner race fault is displayed in Figure 9. Envelope demodulation is then applied to the object signal and the compressed data set, with only 300 samples, tested to determine detectability of fault frequencies.

**Figure 10:**
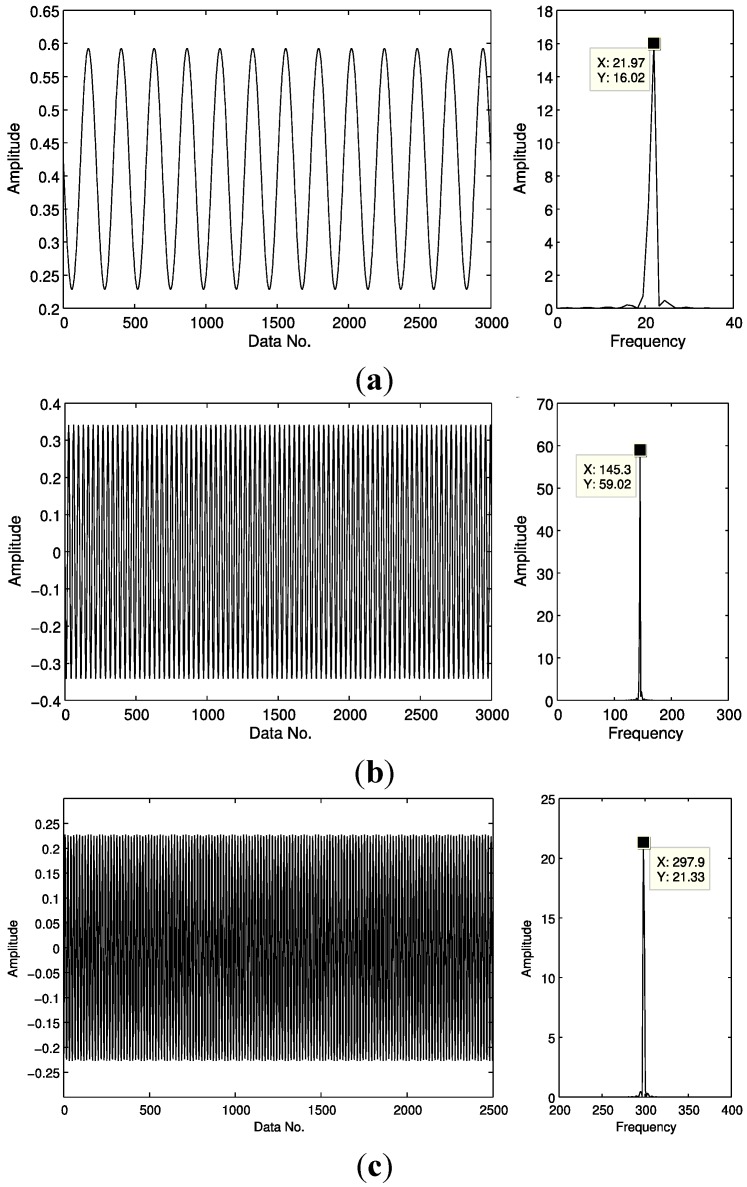
Harmonics detected and their frequencies from envelope samples of the signal shown in Figure 9: (**a**) the first harmonic and its frequency; (**b**) the third harmonic and its frequency; (**c**) the fifth harmonic and its frequency.

Assuming the sparsity *K* = 2, (Figure 10), the first, third and fifth harmonic waves are detected. Frequency of the first detected harmonic component is 21.97 Hz, equal to the theoretical rotational frequency. Frequency of the third detected harmonic component is 145.3 Hz, equal to the inner race fault frequency. Frequency of the fifth detected harmonic component is approximately 290.6 Hz, or, twice the inner race fault frequency, indicating the presence of a fault on the inner race of the bearing.

### 5.3. Comparisons between Different Sampling Rates

**Figure 11:**
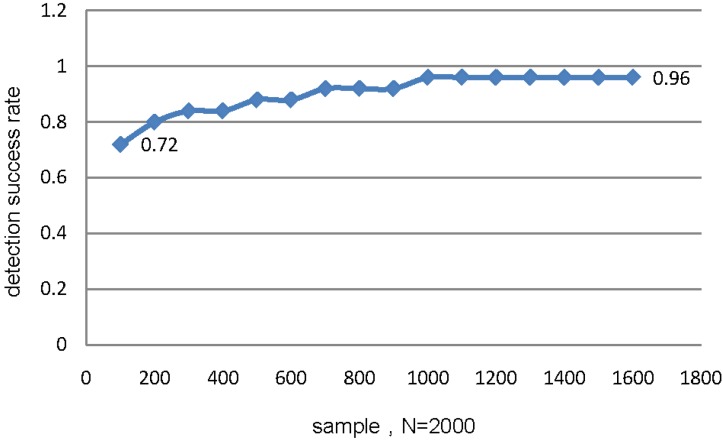
Probability of the compressive fault detection.

A series of experiments with different sampling rates are performed to compare the results with compressed detection probability with different sampling rates. A statistical chart illustrating detection probability *versus* sampling rate is presented in Figure 11. A signal section with samples *N* = 2000 is selected and detection probabilities recorded when the compressed samples vary from 100 to 1600, *i.e.*, the sampling rate varies from 5% to 80%. Detection probability ranging from 72% to 96% may be achieved with the proposed strategy as demonstrated in Figure 11. Variations of detection rates increase as the sampling rate increases until the sampling rate attains up to 50%. The detection rate then stabilizes and nearly approximates the common methods with full samples. The proposed method is not dependent on sampling rate and an acceptable detection probability may be achieved while substantially reducing data acquisition.

## 6. Conclusions

A compressive sensing strategy for detecting roller bearing faults through harmonic waves is proposed in this paper. Fault features may be directly detected from only a few samples without complete reconstruction. During the sampling and iteration process, adequate vital information may be retrieved with limited samples. The corresponding resonance frequency may be detected then by a given sparsity of the potential harmonic wave, thus relaxing requirements for sampling rates required for measurements. Reconstruction and detection may proceed simultaneously without complete recovery, significantly improving detection efficiency as validated by simulations and experiments.
